# Healthcare providers’ perspectives on integrating trauma-informed care for intimate partner violence in HIV/STI clinical settings in Trinidad and Tobago

**DOI:** 10.1371/journal.pgph.0006534

**Published:** 2026-06-23

**Authors:** N. Lyons, M. Olivier, W. Samaroo-Francis, I. Seeram, B. Bhagwandeen, J. Edwards

**Affiliations:** 1 Medical Research Foundation of Trinidad and Tobago, Port of Spain, Trinidad and Tobago; 2 Center for Community Innovation and Research, Port of Spain, Trinidad and Tobago; PLOS: Public Library of Science, UNITED STATES OF AMERICA

## Abstract

Intimate Partner Violence (IPV) remains a significant public health concern, particularly for persons living with HIV (PLHIV) and other sexually transmitted infections (STIs). The intersection of IPV and HIV/STIs creates complex care needs requiring trauma-informed, contextually grounded responses. This study explored healthcare practitioners’ perspectives on existing practices and opportunities to strengthen IPV-related care in public HIV/STI clinical settings in Trinidad and Tobago. Structured weekly consultations were conducted over seven weeks with 13 healthcare practitioners—including physicians, nurses, psychologists, social workers, and peers living with HIV—across five public HIV/STI clinics. Guided by national clinical protocols, discussions explored current practices, care gaps, and system-level challenges. Trauma-Informed Care (TIC) was examined through four core assumptions: realizing the impact of trauma, recognizing its signs, responding appropriately, and resisting re-traumatization. Practitioners also considered how TIC could be integrated *along the care pathway* at five key points: awareness, identification, first-line response, survivor-centered care, and inter-agency referral. Participants identified critical system-level gaps, including the lack of standardized IPV protocols, limited documentation, concerns about privacy, fragmented referral systems, and stigma related to both HIV and IPV. Time constraints and limited training were cited as barriers to consistent, high-quality care. Practitioners also highlighted the elevated vulnerability of women, youth living with HIV, and men who have sex with men to IPV. There was strong consensus on the need for structured TIC training for all levels of staff. The findings underscore urgent opportunities to enhance clinical responses to IPV through the integration of trauma-informed care along the HIV/STI service pathway. Prioritizing TIC can improve care quality, reduce re-traumatization, and strengthen engagement and resilience among populations most at risk.

## Introduction

Intimate Partner Violence (IPV) is a widespread and deeply entrenched public health issue with significant implications for People Living with HIV (PLHIV) and those affected by other sexually transmitted infections (STIs). Global evidence indicates that IPV prevalence is substantially higher among PLHIV than in the general population, with pooled estimates around 15.4%, and higher rates reported in regions such as sub-Saharan Africa and Latin America [1,2]. IPV contributes to adverse health outcomes, including poor mental health, reduced adherence to antiretroviral therapy (ART), increased risk of HIV/STI acquisition and transmission, and limited engagement with healthcare services [1,3]. Beyond women, other populations—including men who have sex with men (MSM), transgender persons, and youth—experience heightened vulnerabilities to IPV and gender-based violence (GBV), particularly within contexts of HIV/STI risk. In the Caribbean, these groups often face overlapping risks due to stigma, discrimination, and marginalization, which can undermine access to healthcare and increase vulnerability to both violence and infection [3,4].

In Trinidad and Tobago, IPV and GBV prevalence remains a critical concern. The National Women’s Health Survey found that approximately 30% of women aged 15–64 had experienced some form of IPV during their lifetime, with substantial consequences for mental and physical health [5]. Recent national guidelines and qualitative studies also point to increasing recognition of violence among MSM and transgender populations, though data remain limited [3,4]. These intersecting vulnerabilities underscore the urgent need for tailored, trauma-informed interventions across diverse populations accessing HIV/STI services.

Given the intersection of HIV vulnerability and IPV risk, HIV/STI clinics represent critical entry points for early identification and response to IPV. Addressing IPV in these settings requires integrated approaches that acknowledge and respond to the physical, psychological, and social dimensions of IPV. This approach is essential not only to improve clinical outcomes and treatment adherence but also to protect dignity, reduce IPV-related trauma, and enhance overall well-being among affected individuals [6,7].

Two complementary frameworks—the Substance Abuse and Mental Health Services Administration’s (SAMHSA) Trauma-Informed Care (TIC) model and the World Health Organization’s (WHO) LIVES approach—offer structured strategies for responding to survivors of IPV. TIC provides an organizational framework that integrates an understanding of trauma’s widespread impact into policies, procedures, and practices designed to promote healing and prevent re-traumatization. SAMHSA identifies six core principles of TIC: safety; trustworthiness and transparency; peer support; collaboration and mutuality; empowerment, voice, and choice; and attention to cultural, historical, and gender issues [8]. Similarly, the WHO LIVES framework guides first-line support by emphasizing active listening, needs assessment, validation, ensuring safety, and supporting empowerment [9].

While the LIVES framework has been increasingly applied in healthcare settings to provide first-line support to IPV survivors, the integration of Trauma-Informed Care (TIC) to address trauma’s psychological impacts and improve patient engagement within HIV/STI clinical contexts remains underexplored. Studies demonstrate TIC’s benefits in similar settings: integration into HIV services for refugee women in the United States improved trust, IPV disclosure, and mental health support [10], while TIC in primary healthcare for women with trauma histories improved engagement, retention, and reduced depression and PTSD symptoms [11]. These findings highlight TIC’s potential to address both physical and psychological health needs, improving outcomes for women experiencing IPV, especially in HIV care contexts.

In Trinidad and Tobago, HIV and STI services are primarily delivered through the decentralized public healthcare system under Regional Health Authorities (RHAs), which provide free HIV testing, counseling, prevention, and treatment services [12,13]. Despite free access, these services face challenges including understaffing, high patient volumes, and infrastructural constraints. STI services are similarly provided through two main centers and satellite clinics [14,15]. This study explored healthcare practitioners’ perspectives on care delivery gaps and survivor-centered support for clients affected by or at risk of IPV in HIV/STI settings. The analysis was guided by the four foundational assumptions of TIC: realizing trauma’s widespread impact, recognizing its signs and symptoms, responding with trauma-informed practices, and resisting re-traumatization. These assumptions framed the organization and interpretation of findings aimed at supporting the development of contextually appropriate and sustainable TIC approaches for IPV response within clinical systems in Trinidad and Tobago.

## Methods

### Ethics statement

The study protocol, data collection instruments and informed consent form were approved by the Campus Research Ethics Committee of the University of the West Indies, St. Augustine, Trinidad, and approval number CREC-SA.2453/12/2023. Consent was obtained verbally from all participants.

### Study design

Between October and November 2023, a series of structured weekly consultations were conducted with a multidisciplinary cohort of healthcare practitioners working in publicly funded HIV/STI clinical settings across Trinidad and Tobago. Over a seven-week period, 13 participants were engaged, including medical doctors, nurses, a psychologist, social workers, and a peer living with HIV (PLHIV). These practitioners were directly involved in frontline service delivery and represented diverse professional perspectives critical to understanding the integration of intimate partner violence (IPV) care into HIV/STI services. The primary objective of the consultations was to explore the identification and management of IPV within HIV/STI clinical settings, with a particular focus on alignment with national IPV guidelines and trauma-informed care (TIC) frameworks. Prior to participation, all individuals were informed of the study’s purpose, and verbal consent was obtained. No personally identifying information was collected, and strict confidentiality was maintained throughout the process in keeping with ethical standards for qualitative inquiry.

The process was initiated with two foundational knowledge-sharing sessions. In Week 1, participants received a presentation on the *National Clinical and Policy Guidelines for the Intimate Partner Violence and Sexual Violence (SV)*. Although the guidelines address both IPV and SV, all subsequent discussions were intentionally focused on IPV. In Week 2, a comprehensive presentation on Trauma-Informed Care (TIC) was delivered. This session introduced participants to both the six core TIC principles—safety, trustworthiness, peer support, collaboration, empowerment, and cultural/gender responsiveness—and the four foundational assumptions as outlined by SAMHSA.

1. Realize the widespread impact of trauma;
2. Recognize the signs and symptoms of trauma in clients and staff;
3. Respond by integrating trauma-informed practices into care systems; and
4. Resist re-traumatization through client-centered, respectful engagement.

These principles and assumptions provided a shared conceptual foundation for all participants and framed the discussions that followed over the remaining weeks. From Week 3 onward, sessions were structured around steps in the pathway of care derived from the national clinical and policy guidelines for IPV and SV. These included,

1. Awareness and identification of IPV;
2. Provision of first-line support;
3. Survivor-centered care, including psychosocial support; and
4. Interagency referral systems to ensure continuity of care and protection.

Each session lasted approximately 2.5 to 3 hours and followed a semi-structured discussion m format. This format balanced guided inquiry with space for open discussion and reflection, allowing for the emergence of context-specific insights and experiences. The following thematic areas were explored:

- Familiarity with and implementation of national IPV protocols
- Existing practices for identifying and supporting survivors
- Barriers to trauma-informed IPV integration, including time constraints, limited staff training, and confidentiality concerns
- Enablers for TIC implementation, such as leadership support and staff motivation4

### Data collection and analysis

Detailed notes were taken during all sessions. The data were analysed using thematic analysis, allowing for the identification of recurring patterns, challenges, and proposed solutions. Coding and analysis were explicitly guided by the four foundational TIC assumptions, which served both as an analytical lens and a structuring framework for interpretation along with the six principles of TIC. Findings from the consultation series were synthesized and are presented in the results and discussion sections of this paper. They offer practical recommendations for strengthening IPV response in HIV/STI services, with particular attention to embedding trauma-informed, survivor-centered care into clinical practice in resource-constrained settings.

## Results

Healthcare practitioners identified a range of systemic, organizational, and clinical barriers that compromised the effective identification and management of intimate partner violence (IPV) within HIV/STI service delivery settings. These challenges revealed significant disconnects between current practices and both national clinical guidelines and trauma-informed care (TIC) principles. Across all interviews, participants expressed a strong desire to align service delivery with both survivor-centered and trauma-aware approaches. Their insights reflected not only the four foundational TIC assumptions—realize, recognize, respond, and resist re-traumatization—but also the six core TIC principles: safety, trustworthiness and transparency, peer support, collaboration and mutuality, empowerment, voice, and choice, and cultural, historical, and gender responsiveness.

### Barriers to effective IPV identification and response

1 Absence of Operational Guidelines

Participants highlighted the lack of standardized clinical protocols for IPV screening, documentation, and response. While national policy frameworks address sexual violence, formal procedures specific to IPV within routine HIV/STI care were reportedly underdeveloped or not implemented. This gap created inconsistencies in care, contributing to uncertainty among staff and fragmented client experiences. The absence of clear, transparent processes undermined trust and limited the perceived safety of disclosing IPV.

2 Weak Referral Pathways

Healthcare providers reported informal, inconsistent referral practices and poor coordination across psychosocial, legal, and community-based services. Without defined referral protocols, clients often received delayed or incomplete support. This undermined collaboration and continuity of care, particularly for vulnerable groups such as LGBTQI+ clients, and migrants,

3 Inadequate Documentation

Poor IPV case documentation emerged as a significant limitation, leading to underreporting and poor tracking of trends. This hindered efforts to monitor prevalence among high-risk subpopulations—such as newly diagnosed HIV/STI clients, pregnant women, and marginalized groups—and to provide targeted interventions. Participants emphasized the need for standardized, trauma-aware documentation practices that are respectful, nonjudgmental, and uphold client confidentiality.

4 Limited Staff Training and Capacity

Practitioners reported limited formal training on IPV and trauma-informed approaches. Most felt ill-equipped to initiate sensitive conversations or respond empathetically to disclosures. This lack of preparedness eroded provider confidence and contributed to missed opportunities to identify and support survivors. Training gaps also impacted staff’s ability to recognize trauma responses, apply culturally responsive care, and avoid re-traumatization.

5 Limited Privacy in Clinical Spaces

Physical space constraints in many clinics compromised privacy and confidentiality. Participants observed that clients often withheld information about IPV due to fear of being overheard or concerns about stigma. The lack of private, safe environments discouraged disclosure and undermined the therapeutic relationship. These limitations clashed directly with TIC principles of safety and trustworthiness.

6 Fast-Paced Clinical Environments

High client volumes and time-limited consultations further constrained providers’ ability to engage in meaningful, trauma-informed care. Without dedicated staff to address violence-related concerns, IPV was often deprioritized or addressed superficially. This resulted in a task-oriented approach that which sometimes conflicted with principles of empowerment, mutuality, and voice and choice.

7 Compounded Stigma among LGBTQI+ Clients

Providers noted that LGBTQI+ individuals were disproportionately affected by stigma, both in healthcare and community settings. This heightened their vulnerability to IPV and contributed to fear of judgment and reduced help-seeking. Practitioners expressed concern that health systems were not adequately responsive to the intersectional needs of LGBTQI+ clients, limiting culturally affirming and trauma-aware care.

### Recommendations for integrating trauma-informed care

Participants strongly endorsed the integration of trauma-informed care into HIV/STI services as a strategy to enhance the identification, response, and overall well-being of clients affected by IPV. Their feedback reflected all four foundational TIC assumptions and operationalized the six core TIC principles. These are summarized in Table 1 and further described below.

**Table 1:** Practitioner feedback and recommendations for trauma-informed care (TIC) integration in HIV/STI clinical settings.

TIC Assumption	Feedback from Practitioners	Recommendations	TIC Principles Reflected
Realize	Limited awareness of trauma’s impact on health-seeking behaviors.	Embed TIC awareness and IPV-specific training across all levels of clinical staff. Develop protocols grounded in TIC.	Safety, Trustworthiness, Cultural Responsiveness
Recognize	Weak IPV detection due to inadequate training and limited private spaces.	Improve clinical inquiry and screening. Enhance confidentiality by redesigning physical spaces.	Safety, Empowerment, Cultural Responsiveness
Respond	Informal referral pathways and lack of coordination across sectors. Need for peer-led interventions.	Strengthen first-line response using the LIVES+TIC model. Establish structured referral systems. Support peer IPV navigators, including PLHIV.	Collaboration, Peer Support, Empowerment
Resist Re-traumatization	Risk of harm from stigmatizing responses and insensitive documentation.	Train staff in trauma-sensitive communication. Implement respectful, standardized documentation practices. Promote TIC-based health promotion and prevention strategies.	Trustworthiness, Safety, Voice and Choice

### Application of TIC assumptions and principles

#### Assumption I: Realize — Building awareness and shared understanding.

Practitioners underscored the need for comprehensive training to foster awareness of how trauma and IPV affect client behaviors, health outcomes, and treatment adherence. Embedding TIC at the organizational level was viewed as foundational to creating a consistent and trustworthy care environment. This realization must also include understanding the historical and cultural context of trauma among marginalized populations, including LGBTQI+ persons and migrants accessing HIV/STI services.

#### Assumption II: Recognize — Identifying and responding to trauma.

Participants highlighted the importance of strengthening clinical inquiry and improving case documentation. Creating safer environments—both physical and interpersonal—was identified as essential to encouraging disclosure. Confidential, non-judgmental communication strategies and culturally responsive approaches were considered critical for enabling recognition of trauma without causing further harm.

#### Assumption III: Respond — Strengthening systems and support.

Recommendations emphasized the development of structured, multi-sectoral referral pathways that connect clients to appropriate legal, psychosocial, and community-based services. Practitioners also supported the inclusion of peer navigators—particularly persons living with HIV—within care teams to build trust and reduce stigma. This collaborative approach fosters empowerment and reinforces continuity of care.

#### Assumption IV: Resist re-traumatization — Fostering healing, not harm.

To prevent re-traumatization, practitioners advocated for person-centered, empathetic engagement rooted in survivor choice and agency. Standardizing documentation practices with a trauma-aware lens, reducing stigmatizing language, and promoting clinic-wide health promotion strategies were seen as essential steps toward creating a healing clinical culture that upholds safety and respect.

## Discussion

This study highlights critical systemic and operational gaps in the provision of care for intimate partner violence (IPV) survivors and persons with elevated risks within HIV/STI clinical settings in Trinidad and Tobago. Despite extensive global evidence linking IPV with poor HIV-related health outcomes—including decreased antiretroviral therapy (ART) adherence, increased transmission risk, and deteriorated mental health [1,16,17]—IPV remains insufficiently addressed in HIV/STI care delivery settings. Consistent with international research, healthcare practitioners in this study reported persistent structural and institutional barriers that impede IPV screening, documentation, and referrals. These studies collectively examine how stigma and systemic barriers impact disclosure and care in sensitive health contexts. Turan et al. [18] found that internalized and anticipated HIV-related stigma significantly reduce healthcare engagement and psychological well-being among people living with HIV. Furniss et al. [19] identified fear, shame, and distrust as major barriers to abuse disclosure in clinical settings. Similarly, Sprague et al. [20] highlighted key obstacles to intimate partner violence screening, including provider discomfort, lack of training, and institutional constraints. These constraints are often exacerbated in high-volume clinical settings where time pressures limits providers’ ability to engage in sensitive, client-centered care.

Stigma related to HIV status and sexual identity creates significant and multifaceted barriers to intimate partner violence (IPV) care among men who have sex with men (MSM) and other LGBTQI+ populations. Multiple studies collectively demonstrate that internalized homophobia, anticipated stigma, and broader minority stress contribute to profound feelings of fear, shame, and social isolation. These psychological burdens discourage individuals from disclosing experiences of abuse or seeking supportive services, thereby perpetuating cycles of violence and unmet health needs [21–23]. While stigma is consistently identified as a critical barrier to healthcare engagement across populations, some research highlights additional intersecting factors that intensify these challenges. For example, MSM and other LGBTQI+ individuals living with HIV or other sexually transmitted infections (STIs) experience compounded stigma related to both their health status and sexual identity, which further reduces access to IPV-related services and support [24,25]. Individuals living with HIV also encounter stigma related to their health status, which increases vulnerability to IPV and reduces the likelihood of help-seeking behaviors [24]. This intersectional stigma not only affects individuals’ mental health and social support but also influences systemic responsiveness, as healthcare systems and IPV interventions may lack cultural competence or fail to address the unique needs of LGBTQI+ clients. Taken together, these findings underscore the urgent need for trauma-informed, stigma-sensitive IPV interventions that are explicitly designed to address the complex, intersecting social identities and experiences faced by LGBTQI+ individuals, to improve disclosure, engagement, and health outcomes.

This study applied the Trauma-Informed Care (TIC) framework—which is based on four foundational assumptions (realize, recognize, respond, and resist re-traumatization)—to guide analysis and interpretation of practitioner feedback. These assumptions, combined with six core TIC principles—safety, trustworthiness and transparency, peer support, collaboration and mutuality, empowerment, and cultural/historical/gender responsiveness—provided a robust conceptual model to identify actionable strategies for improving IPV response in clinical settings.

1 **Realize: Acknowledging the Widespread Impact of Trauma**

Practitioners emphasized the importance of organizational awareness regarding trauma’s impact on health-seeking behaviors and care engagement. The absence of operational guidance for IPV screening and response reflected a system-wide under-recognition of trauma’s role in shaping client experiences. This aligns with ongoing recommendations for healthcare institutions to develop trauma-informed policies and provide training for all staff, thereby promoting shared understanding and enhancing trust between clients and providers [26,27].

2 **Recognize: Improving Identification and Safe Disclosure**

A major finding of this study was the inadequate identification of IPV due to limited clinical training, weak documentation systems, and insufficient privacy in service environments. These issues contributed to missed opportunities to support survivors—particularly those facing intersectional stigma, such as LGBTQI+ persons and migrants. The recommendation to create confidential spaces for sensitive inquiry is supported by global evidence showing that physical and emotional safety is a cornerstone of effective IPV disclosure and intervention [9,29]. Culturally responsive engagement and non-judgmental communication were also emphasized to support equitable care.

3 **Respond: Coordinating Multi-Sectoral and Peer-Supported Interventions**

Practitioners strongly advocated for the development of clear, multi-sectoral referral pathways linking clinical care with psychosocial, legal, and community-based support. The lack of formal systems impeded continuity of care and increased the burden on survivors to navigate complex service environments. Recommendations to adopt WHO’s LIVES model—with trauma-informed adaptations—and to integrate peer support, particularly through people living with HIV (PLHIV) as IPV patient peers, reflects best practices for fostering client trust and empowerment [29]. Peer support promotes mutuality, reduces stigma, and improves care continuity, particularly in under-resourced settings.

4 **Resist Re-traumatization: Fostering Healing and Survivor Agency**

Participants described how stigmatizing provider behaviors, insensitive documentation, and rushed consultations risked re-traumatizing clients. These findings support broader literature that identifies non-judgmental, trauma-sensitive engagement and standardized, respectful documentation practices as key to creating healing environments [26–28]. Training in empathetic communication, the use of simplified screening tools, and survivor-centered prevention education were proposed as strategies to shift clinic culture toward resilience and recovery.

While trauma-informed approaches have gained traction in addressing gender-based violence among women, this study underscores the need to expand TIC application to other vulnerable populations—particularly men who have sex with men (MSM), transgender individuals, and sex workers—who experience elevated HIV risk and are often excluded from IPV interventions. The diverse trauma histories of these populations—including childhood sexual abuse, community violence, and poly victimization—require tailored TIC adaptations that go beyond gender binaries and traditional IPV frameworks. Yet, existing literature provides minimal guidance on how to operationalize TIC for these groups, especially within HIV care contexts [30]. Addressing this gap requires inclusive program design, participatory research, and the synthesis of emerging evidence from low- and middle-income countries.

This study contributes to the limited literature on trauma-informed care in Caribbean public health systems. Unlike much of the existing evidence drawn from high-income or sub-Saharan African contexts, this research centers the lived experiences of multidisciplinary healthcare providers working in resource-constrained HIV/STI clinics in a small island developing state. By incorporating the perspectives of doctors, nurses, psychologists, social workers, and peer navigators, the study surfaces both structural limitations and interpersonal dynamics that shape IPV response in real-world practice. The findings complement global guidance, such as the WHO Clinical and Policy Guidelines, by grounding them in a culturally specific, intersectionality-informed context. This is essential for ensuring that TIC frameworks are not only clinically effective but socially relevant.

In summary, this study demonstrates the feasibility and value of integrating TIC into HIV/STI service delivery in Trinidad and Tobago. Even in low-resource settings, targeted strategies such as staff sensitization, peer navigation, referral strengthening, and clinic redesign can foster safety, enhance survivor engagement, and mitigate the long-term consequences of trauma. The principles of trauma-informed care offer a transformative, equity-driven framework for building responsive health systems. However, there remains a need for implementation research to evaluate the effectiveness, acceptability, and sustainability of TIC models within HIV/STI settings, particularly among underserved populations.

### Study strengths and limitations

A key strength of this study lies in its inclusion of 13 healthcare practitioners across diverse disciplines within publicly funded HIV/STI clinical settings in Trinidad and Tobago. This multidisciplinary composition—which included physicians, nurses, a psychologist, social workers, and a peer living with HIV—enabled the capture of varied practitioner perspectives and experiences, enhancing the contextual relevance and richness of the findings. The use of structured weekly consultations over a seven-week period facilitated in-depth exploration of both systemic and practice-level challenges related to IPV care, while also allowing time for reflection and discussion grounded in national clinical guidelines and trauma-informed care (TIC) frameworks.

However, several limitations must be acknowledged. First, findings are specific to the healthcare system and sociocultural context of Trinidad and Tobago and may not be directly generalizable to other Caribbean nations without local contextual adaptation. Second, the data were collected through self-reported reflections, which may be subject to social desirability or recall bias.

Third, the study did not include the perspectives of IPV survivors or clients accessing HIV/STI services. This absence may have excluded critical insights into lived experiences, disclosure barriers, and unmet needs. Lastly, while this study applies the four foundational TIC assumptions—realize, recognize, respond, and resist re-traumatization—as an analytical framework, it does not assess the outcomes of TIC implementation, nor does it directly address broader structural constraints, such as funding availability, workforce shortages, or policy-level reform. These gaps represent important directions for future research and implementation studies.

## Conclusion

In summary, this study identifies critical service delivery gaps and presents actionable, context-specific recommendations for integrating trauma-informed care into HIV/STI service settings in Trinidad and Tobago. The findings provide a foundation for policy reform, workforce training, and inter agency collaboration, and support the evolution of a public health system that is more responsive to the needs of IPV survivors and aligned with principles of safety, equity, and resilience.

The integration of TIC principles into HIV/STI care—including revised protocols, routine IPV screening, staff sensitization, robust referral pathways, and the inclusion of PLHIV peer navigators—can foster safer, more inclusive clinical environments. These strategies are particularly vital for highly stigmatized populations, such as LGBTQI+ individuals, who often face compounded barriers to care. Implementing TIC in conjunction with established frameworks, such as the WHO LIVES model, holds the potential to improve health outcomes, increase IPV disclosure, minimize re-traumatization, and promote sustained engagement in care.

This study underscores the urgent need to strengthen IPV response mechanisms within HIV/STI clinical settings in Trinidad and Tobago. Despite the well-documented relationship between IPV, HIV/STI vulnerability, and adverse treatment outcomes, IPV remains insufficiently integrated into service delivery models. Using the TIC framework—particularly its four assumptions of realize, recognize, respond, and resist re-traumatization—this study highlights concrete, feasible opportunities for system-level transformation. Moreover, this work contributes meaningfully to the growing evidence base on trauma-informed health systems, especially within resource-constrained and small island developing states. Future research should prioritize the design, piloting, and scale-up of TIC interventions in Caribbean contexts, with measurable outcomes such as improved patient safety, ART adherence, service utilization, and psychosocial wellbeing. Given the elevated risks faced by women, MSM, youth, and other populations affected by HIV/STIs, it is clear that single-level interventions are no longer sufficient. Trauma-informed initiatives must therefore be developed in ways that address structural and systemic drivers of health inequity, in order to sustainably improve outcomes for vulnerable populations.

## Supporting information

S1 Dataset Summary of thematic data and de-identified participant excerpts. (DOCX)
