# Supplementary material for: Healthcare providers’ perspectives on integrating trauma-informed care for intimate partner violence in HIV/STI clinical settings in Trinidad and Tobago
Source: PLOS Glob Public Health. 2026 Jun 23;6(6):e0006534. doi: 10.1371/journal.pgph.0006534 (PMC13289925; doi:10.1371/journal.pgph.0006534)
Supplement: S1 Dataset — (DOCX) [file pgph.0006534.s001.docx]

**Summary of Thematic Data and De-identified Participant Excerpts**

| TIC Assumption / Theme | Subtheme | Description / Analytical Summary | Illustrative Excerpt (De-identified) | Participant Code |
| --- | --- | --- | --- | --- |
| **REALIZE – Building Awareness and Shared Understanding** | Limited awareness of trauma’s impact | Practitioners noted that trauma and IPV are often treated as separate from HIV care, with little awareness of how they affect adherence and engagement. | “We don’t always ask about violence unless it’s obvious. I think we underestimate how trauma affects how people come for treatment.” | P03 |
| **RECOGNIZE – Identifying and Responding to Trauma** | Weak IPV detection practices | Providers reported difficulty initiating conversations about IPV due to limited training and privacy constraints. | “Even if we suspect abuse, it’s hard to bring it up when others can overhear.” | P07 |
| **RESPOND – Strengthening Systems and Support** | Informal and fragmented referral pathways | Participants described inconsistent referral processes and a lack of follow-up between services. | “Sometimes we just give a number to call, but there’s no feedback or connection after that.” | P09 |
| **RESPOND – Strengthening Systems and Support** | Need for peer navigators | Providers believed that trained peers living with HIV could bridge trust gaps and support disclosure. | “When clients talk to someone who’s been through it, they open up more easily.” | P02 |
| **RESIST RE-TRAUMATIZATION – Fostering Healing, Not Harm** | Risk of stigmatizing responses | Some staff responses to IPV disclosures were unintentionally dismissive or judgmental. | “Clients have said they feel blamed when they talk about abuse—like it’s their fault for staying.” | P10 |
| **RESIST RE-TRAUMATIZATION – Fostering Healing, Not Harm** | Need for trauma-sensitive documentation | Inconsistent or judgmental documentation practices created fear of disclosure. | “People worry about what’s written in their files… whether it could be used against them.” | P05 |
